# *Cordyceps guangdongensis* lipid-lowering formula alleviates fat and lipid accumulation by modulating gut microbiota and short-chain fatty acids in high-fat diet mice

**DOI:** 10.3389/fnut.2022.1038740

**Published:** 2022-11-02

**Authors:** Gangzheng Wang, Chengyuan Sun, Bojun Xie, Tao Wang, Hongwei Liu, Xianglian Chen, Qiuju Huang, Chenghua Zhang, Taihui Li, Wangqiu Deng

**Affiliations:** ^1^Guangdong Provincial Key Laboratory of Microbial Culture Collection and Application, State Key Laboratory of Applied Microbiology Southern China, Institute of Microbiology, Guangdong Academy of Sciences, Guangzhou, China; ^2^State Key Laboratory of Mycology, Institute of Microbiology, Chinese Academy of Sciences, Beijing, China; ^3^Hunan Provincial Key Laboratory for Synthetic Biology of Traditional Chinese Medicine, Hunan University of Medicine, Huaihua, China

**Keywords:** obesity, *Bacteroides* and *Bifidobacterium*, short-chain fatty acids, association analysis, *Cordyceps guangdongensis* lipid-lowering formula

## Abstract

Obesity has caused serious health and economic problems in the world. *Cordyceps guangdongensis* is a high-value macrofungus with broad application potential in the food and bio-medicine industry. This current study aimed to estimate the role of *C. guangdongensis* lipid-lowering compound formula (CGLC) in regulating fat and lipid accumulation, gut microbiota balance, short-chain fatty acid (SCFA) contents, and expression levels of genes involved in fat and lipid metabolism in high-fat diet (HFD) mice. The results showed that CGLC intervention markedly reduced body weights and fat accumulation in HFD mice, improved glucose tolerance and blood lipid levels, and decreased lipid droplet accumulation and fat vacuole levels in the liver. CGLC decreased the ratio of Firmicutes and Bacteroidetes and increased the relative abundances of *Bacteroides* (*B. acidifaciens*) and *Bifidobacterium* (*B. pseudolongum*). In addition, CGLC treatment significantly promoted the production of SCFAs and regulated the relative expression levels of genes involved in fat and lipid metabolism in liver. Association analysis showed that several species of *Bacteroides* and most of SCFAs were significantly associated with serum lipid indicators. These results suggested that CGLC is a novel candidate formulation for treating obesity and non-alcohol fatty liver by regulating gut microbiota, SCFAs, and genes involved in fat and lipid metabolism.

## Introduction

According to the World Health Organization (WHO) report in 2021, more than 650 million adults were obese, and over 340 million children and adolescents aged 5–19 were overweight or obese in 2016. The global obesity rate in 2030 will likely reach 20% of the adults (1). Increasing evidence indicates that long-term uncontrolled obesity can lead to various many metabolic disorders, such as hyperlipidemia, type 2 diabetes mellitus (T2MD), non-alcohol fatty liver disease (NAFLD), chronic kidney disease, cancers, and cardiovascular disease (2–5). These reports indicate that obesity has become a worldwide public health problem and has caused a rapid increase in healthcare expenditures for both individuals and the society in general. Several studies have shown that the structure of gut microbiota between obese individuals and healthy individuals showed an obvious difference (6, 7). When the gut microbiota from the obese mice were transferred into the healthy mice, both the energy absorption and body weights increased for the healthy mice (8). A growing body of evidence has shown that gut microbiota could regulate the production of short-chain fatty acids (SCFAs), bile acids, and the expression of genes related to lipid metabolism and immune response, further affecting glucose and insulin tolerance, hyperlipidemia, energy metabolism, and inflammation (9–12).

Traditional Chinese medicine (TCM) has a long application history in the treatment of hyperlipidemia and hypertension in many East Asian regions. Many reports showed that TCM decreased the symptoms of obesity and many metabolism disorders. These studies have revealed that the active ingredients in TCM can influence multiple target regulatory networks, including regulating fat metabolism, promoting the production of SCFAs, restoring the gut microbial dysbiosis, and enhancing hormone level (13–16). For example, *Phaseolus vulgaris*, a popular food in Asia and Eastern country, significantly reduced food intake, body weight gain, lipid accumulation, carbohydrate absorption, and metabolism in obese animals (17, 18). Interestingly, *P. vulgaris* extract significantly increased the relative abundance of *Lactobacillus*, *Bifidobacterium*, and *Akkermansia* at the genus level and further improved insulin resistance in HFD mice (19). Lotus leaves, widely used as a herbal medicine and food in China and other East Asian countries, effectively inhibited fat accumulation, improved dyslipidemia, and alleviated liver injury and inflammation by inhibiting lipase activity, lipogenesis, and adipocyte differentiation (20, 21). *Pueraria lobata* root and green tea, which developed into functional foods in East Asia, showed an beneficial effect on reducing fat weight increase, lipid accumulation in liver, and blood lipid indicators (22–25). Hence, the research and development of TCM for the prevention and treatment of obesity and metabolism disorder diseases is an important area of interest.

*Cordyceps guangdongensis* is a novel Cordyceps edible-medicinal fungus. Its fruiting body showed a similar metabolite profile as *Ophiocordyceps sinensis* and exhibited a notable beneficial effect on anti-virus (influenza virus H9N2), chronic renal failure, and anti-inflammatory diseases (26–28). Recently, we found that ethanol extract of *C. guangdongensis* markedly reduced body weight of high-fat diet mice, improved serum lipid indicators, improved fat vacuolated lesion level in the liver, and reduced the ratio of Firmicutes and Bacteroidetes (29). TCM shows the advantages of having fewer side effects and lower toxicity and cost compared to western medicines (14). Therefore, the above-mentioned materials were collected and mixed with another functional food (conjugated linoleic glyceride ester CLGE and L-arabinose, also prevented obesity) to produce *C. guangdongensis* lipid-lowering compound formula (CGLC).

The present study aimed at evaluating the effects of CGLC on obese mice in high-fat diet from several points, including fat accumulation and serum indicators, the structure of gut microbiota, SCFAs production, and the relative expression levels of genes related to fat and lipid metabolism. In addition, association analyses of several indexes were performed to comprehensively explain the anti-obesity effects of CGLC. Our results will provide more selections of health anti-obesity for the growing obese population.

## Materials and methods

### *Cordyceps guangdongensis* lipid-lowering compound formula preparation

About 100 g CGLC contains 16 g fruiting body powder of *C. guangdongensis*, 16 g CLGE, 16 g L-arabinose, 14 g *Phaseolus vulgaris* Linn. powder, 11 g lotus leaves powder, 8 g root powder of *Pueraria Lobata*, 6 g maltodextrin, 5 g instant green tea powder, 4.3 g microcrystalline cellulose, 2 g magnesium stearate, and 1.7 g coating powder. *C. guangdongensis* fruiting bodies were provided by Guangdong Kehua Huitai Biotechnology Co., LTD, dried in a rotary drier at 55°C, and were ground using ultra-low temperature pulverizer at −20°C. L-arabinose and conjugated linoleic acid glycerides were purchased in Zhengzhou Best Health Technology Co., LTD. Microcrystalline cellulose and magnesium stearate were purchased in Henan Sugar Cabinet Food Co., LTD. Coating powder was purchased in Jiangsu Xinyu Pharmaceutical Co., LTD. The remaining were purchased in Shaanxi Guosheng Biotechnology Co., LTD. All the prepared materials were directly and thoroughly mixed to create CGLC.

### Animals and treatments

Forty-five male C57BL/6J mice (6-week-old, specific pathogen-free SPF), purchased from Shenzhen Saibenuo Biological Technology Co., Ltd (China), were housed in a SPF animal room on 12-h light–dark cycle with 22 ± 1°C temperature and 55 ± 10% relative humidity. Meanwhile, these mice were provided with food and water *ad libitum*. After 1 week of adaptation to regular food and water, all the mice were randomly divided into three groups (*n* = 15): control check group (CK), high-fat diet group (HFD), and CGLC group (CGLC). Mice of CK group were fed with a regular chow diet (Jiangsu Syony Pharmaceutical Bio-engineering Co. LTD, China), while mice in HFD and CGLC groups were fed with a 60% HFD (D12492, SYSE Bio-tech Co., LTD., China). After 8 weeks, mice in the CGLC group received CGLC solution in dose of 1.5 g/kg/d for 14 weeks, whereas mice from CK and HFD group daily were gavaged using 0.8% saline of the same volume as the CGLC group. During the process of gavage experiments, food intake and mouse body weights were weekly documented, and energy intake was calculated by multiplying the energy by grams of food consumed. The calorie values of regular chow diet and HFD were 3.53 and 5.24 Kcal/g. At last, all mice were fasted for 12 h and then were sacrificed by anesthesia. All animal experiments were approved by Animal Care and Use Committee of Institute of Microbiology, Guangdong Academy of Science (No. GT-IACUC20210406).

### Measurement of oral glucose tolerance test and fat mass-related indices

At 21 weeks, all mice were fasted for 12 h and orally administered glucose (1.0 g/kg body weight) to perform the oral glucose tolerance test (OGTT). After glucose administration, the blood glucose levels at 0, 15, 30, 60, 90, and 120 min were monitored using a glucometer (HGM-121, Omron, China) and calculated the area under the curve (AUC). At the end of the experiment, body weights and lengths (from nose apex to anus) were measured to calculate the rate of body weight increase and Lee’s efficiency on the basis of the description in a previous study (30). In addition, the epididymis adipose tissue, inguinal adipose tissue, brown adipose tissue, and liver tissue were weighed and used to calculate the fat mass-related index (tissue weight/body mass weight × 100%).

### Serum index analysis and histological section observation

At the end of the experiment, blood samples of 15 mice per group were collected from thorax after CO_2_ anesthesia. Then, those blood samples were equilibrated at room temperature for 1 h and were centrifuged at 3,000 revolution per minute (rpm) for 15 min at 4°C. The serum was collected and stored at −80°C. Total cholesterol (TC), triglycerides (TG), low-density lipoprotein cholesterol (LDL-C), and the activity of alanine aminotransferase (ALT) in serum were measured using a commercial kit (Nanjing Jiancheng Bioengineering Institute, China). The epididymal adipose and liver tissues were washed with 0.8% saline and fixed in a 4% paraformaldehyde solution. All the tissues were embedded in paraffin and cut into tissue slices with a thickness of 4 μm. Then, these tissues were stained with oil red O and hematoxylin and eosin (HE). Finally, all the slides were observed and photographed using an optical microscope (DM6M, Leica microsystems, Germany).

### Analysis of gut microbiota and short-chain fatty acid contents in feces

At 23 weeks, 12 fresh fecal samples for each group were randomly collected, divided into six mixture samples, and immediately stored in a −80°C freezer to analyze gut microbial composition and SCFA contents in Wuhan Metware Biotechnology Co., Ltd. (Wuhan, China). The concentration and integrity of the extracted DNA by CTAB method were detected using 1.5% (w/v) agarose gel electrophoresis. Then, the V3-V4 region of 16S rRNA (ribosomal RNA) gene was amplified with 515F/806R primers. The purified PCR amplicons were used for library construction using TruSeq^®^ DNA PCR-Free Sample Preparation Kit (FC-121-3001, Illumina, USA) and sequenced on an Illumina Novaseq6000 platform. Paired-end reads were assembled into raw tags using FLASH (Version 1.2.7), and raw tags were filtered using QIIME (V1.9.1) and mapped to species annotation database to obtain effective tags, which were clustered into operational taxonomical units (OUTs) at 97% identity using Uparse algorithm (31).

About 20 mg fecal samples from each mixture were dissolved in 1 mL phosphoric acid (0.5% v/v) solution with a small steel ball and were grinded for 10 min and ultrasonicated for 5 min. After 10 min of centrifugation at 12,000 rpm and 4°C, methyl tert-butyl ether (contain internal standard) solution was mixed with the same volume of supernatant, and the mixture was vortexed for 3 min and ultrasonicated for 5 min. The mixture was then centrifuged for 10 min at 12,000 rpm at 4°C. The supernatant was collected and used for the detection of SCFA contents on the Agilent 7890B-7000D GC-MS/MS platform (32). Every group included six replicates.

### Analysis of transcriptomes in hepatic tissue

Total RNAs of hepatic samples were extracted using a TRIzol Kit (Invitrogen, Dalian, China) according to the manufacturer’s instruction. Every group had four replicates. The qualitative and quantitative analyses of all RNAs were performed on an Agilent 2100 Bioanalyzer (Agilent Technologies, Palo Alto, CA, USA). Then, all the RNAs were used for library construction and were sequenced on an Illumina HiSeq platform. After quality trimming and adapter clipping, all the reads were mapped to the reference genome using Hisat2 v2.0.4. DESeq2 was adopted to identify differential expression genes (DEGs) between groups (33). DEGs involved in cholesterol metabolism, fat metabolism, hepatitis B, non-alcoholic fatty liver, lipolysis in adipocytes, and TNF signal pathway were selected for further analysis.

### Statistical and association analysis

Statistical differences among CK, HFD, and CGLC groups were determined by one-way ANOVA using the Statistical Package for the Social Sciences program (SPSS 22.0). Error bars represent the standard deviation from the mean of six independent replications in every group. Association analyses of many indexes and heat-map figures were performed using SupCorrPlot and Heat-map illustrator packages of TBtools software (34). TBtools software and GraphPad Prism 8 were used for figure construction. Asterisks were used to represent significant differences between each group.

## Results

### *Cordyceps guangdongensis* lipid-lowering compound formula reduced body weights and fat accumulation in high-fat diet mice

**FIGURE 1:**
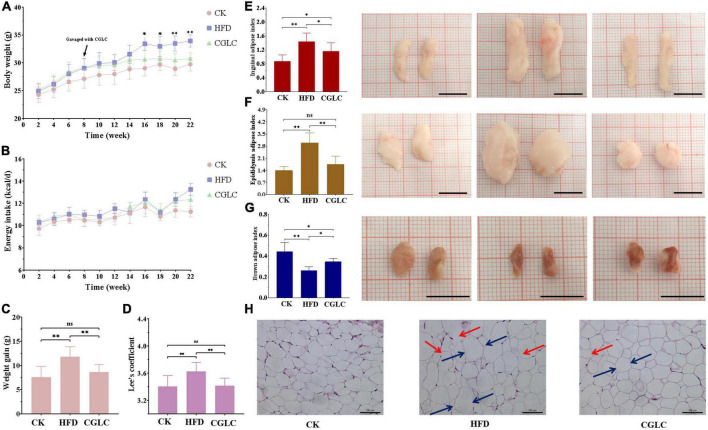
Effects of CGLC on body weight gain, energy intake, fat accumulation, and characteristics in obese mice induced by HFD. Changes in body weight **(A)** and average energy intake **(B)** over 22 weeks. Measurements of body weight gain **(C)** and Lee’s coefficients **(D)**. Effects of CGLC on fat indexes and morphological characteristics of inguinal fat **(E)**, epididymis fat **(F)**, and brown fat **(G)**. Effect of CGLC on epididymis adipose cells [scale bar 100 μm, 200× **(H)**]. ns, no significant difference; *, significant difference (*P* < 0.05); ^**^, highly significant difference (*P* < 0.01); black line, 1 cm; blue arrows, cell diameter; red arrows, cytoplasmic fractures.

At 8th week of the experiment, the obvious differences of body weights were found between CK and another two groups (Figure 1A). After 14-week gavage of CGLC, a highly significant difference of body weights emerged between HFD and CGLC groups (34.43 ± 2.01 vs 30.73 ± 1.29 g). During the process of gavage, the average energy intakes between HFD and CGLC groups had no obvious difference (Figure 1B). Compared with mice in the HFD group, mice in the CGLC group showed a lower increase in body weights (Figure 1C). In addition, Lee’s coefficient of mice in the HFD group (3.63 ± 0.13) was higher than those of CK and CGLC groups (3.40 ± 0.16 vs 3.41 ± 0.09, Figure 1D).

From the perspective of adipose weights, compared with mice in the CK group, mice in the HFD group showed a markedly increase in the inguinal and epididymis adipose indexes (Figures 1E,F), whereas an obvious decrease was found for the brown adipose index at the end of the experiment (Figure 1G). After CGLC administration for 14 weeks, the inguinal and epididymis adipose indexes significantly decreased (*P* < 0.05 and *P* < 0.01), and brown adipose index increased (*P* < 0.05). In terms of morphological characteristics of adipose tissues, the sizes of inguinal and epididymis adipose exhibited 30.99 and 38.10% decreases, respectively, but a 26.47% increase was found for the brown adipose size in width. Depending on microscopic observation of epididymis adipose tissue, compared with mice in the CK group, epididymis adipose cells in the HFD group were irregular, enlarged in diameter (blue arrows), had more cytoplasmic fractures (red arrows), and significantly decreased for cell number in the same field of viewpoint (Figure 1H). However, after 14-week administration of CGLC, epididymis adipose cells were more tightly arranged, showed less cytoplasmic fractures, reduced in diameter, and had an obvious increase in cell number. These results indicated that CGLC can reduce body weight gain and adipose accumulation in HFD mice without suppressing energy intake.

### *Cordyceps guangdongensis* lipid-lowering compound formula improved lipid and fat metabolism in serum and liver of high-fat diet mice

**FIGURE 2:**
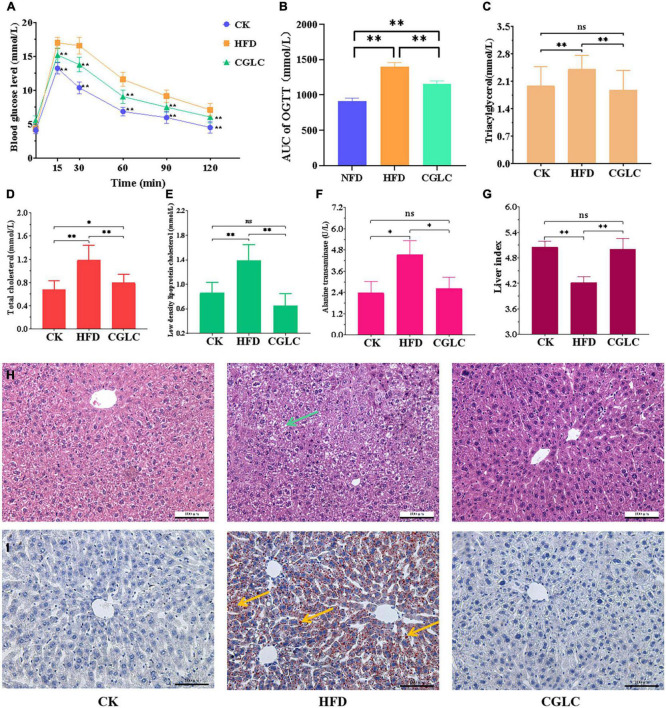
Effects of CGLC on blood glucose, serum lipid levels, and liver tissue. **(A)** OGGT. **(B)** Area under the curve. **(C)** TG levels. **(D)** TC levels. **(E)** LDL-C levels. **(F)** Alanine transaminase activity. **(G)** Liver index. Pathological section observation of liver tissue stained by HE **(H)** and oil red O **(I)**, respectively (scale bar 100 μm, 200×). AUC, area under the curve; green arrow, tiny round fat vacuoles; blue arrows, lipid droplets. ns, no significant difference; *, significant difference (*P* < 0.05); ^**^, highly significant difference (*P* < 0.01).

OGTT results showed that 12-week intervention of CGLC reduced blood glucose levels of HFD mice at 15, 30, 60, 90, and 120 min (Figure 2A, *P* < 0.01), and the area under the curve (AUC) of the CGLC group exhibited 20.66% decrease (Figure 2B, *P* < 0.01), indicating that CGLC improved the glucose tolerance of high-fat diet mice. From the perspective of lipid metabolism, we found that 14-week administration of CGLC significantly decreased the levels of TG, TC, and LDL-C in serum (Figures 2C–E, *P* < 0.01) compared to the HFD group. Among the above three indexes, the levels of TG and LDL-C in the CGLC group had no obvious differences with the CK group and showed, respectively, 21.87 and 52.56% decreases compared to the HFD group (Figures 2C,E). However, the levels of TC in the CGLC group reduced 32.91% of that in the HFD group, whereas the levels were higher than that in the CK group (Figure 2D). These results demonstrated that CGLC can improve blood glucose tolerance and hyperlipidemic level in high-fat diet mice by decreasing the TG, TC, and LDL-C levels in serum (especially TG and LDL-C).

As shown in Figure 2F, the 14-week administration of CGLC reduced the activity of alanine transaminase (ALT) in liver tissue, and a 42.20% decrease level was obtained in the comparison between the CP and HFD groups. At the same time, we found that the liver index in the CGLC group showed a 18.35% increase compared with that in the HFD group (Figure 2G). Based on the observation of liver histological sections stained by HE (Figure 2H), we observed that plenty of fat vacuoles (green arrow) were found in the liver cells of the HFD mice, and their frontier and morphology were obscure; nevertheless, frontier and morphology of liver cells in the CGLC mice were clear and regular, and they had few fat vacuoles. Oil red O staining observation showed that a large amount of lipid drops (red arrows) were deposited in the liver tissue of HFD group mice, and most of the liver tissue area was dark red (Figure 2I). However, CGLC significantly decreased the lipid accumulation compared with the HFD group, and liver tissues in the CGLC group were very slight red. The aforementioned findings revealed that CGLC alleviated lipid accumulation in the liver and further protected the liver cells from fat damage.

### *Cordyceps guangdongensis* lipid-lowering compound formula restored gut microbial dysbiosis in high-fat diet mice

**FIGURE 3:**
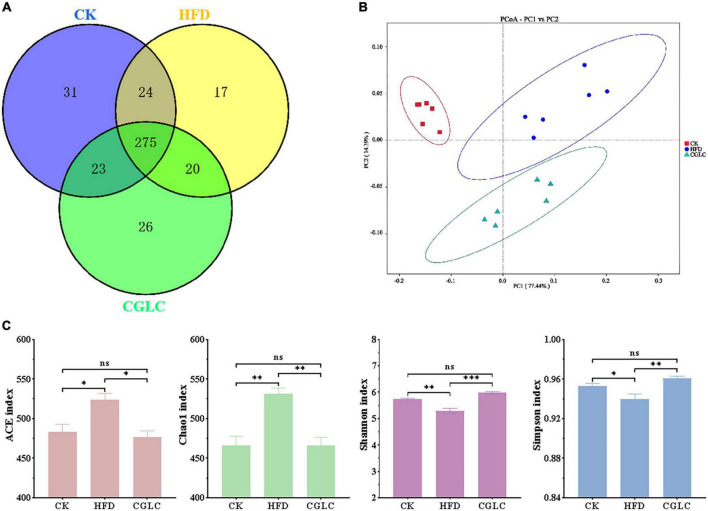
Analyses of CGLC on diversity and richness of the gut microbiota. Venn graph showing the OTU numbers of gut microbiota from three groups **(A)**. Weighted UniFrac PCoA analysis of gut microbiota on the basis of three group OTU data **(B)**. Analysis of alpha-diversity [abundance coverage-based estimator (ACE), Chao1, Shannon, and Simpson indices] in each group **(C)**. ns, no significant difference; *, significant difference (*P* < 0.05), ^**^ and ^***^, highly significant difference (*P* < 0.01 and *P* < 0.001).

A total of 122,468 effective tags were obtained from 18 fecal samples (Supplementary Table 1). On the basis of 97% sequence similarity criterion, a total of 416 OTUs were generated, 353 were in the CK group, 351 were in the HFD group, and 344 were in the CGLC group (Figure 3A), including 31 unique OTUs in the CK group, 17 unique OTUs in the HFD group, and 26 unique OTUs in the CGLC group. PCoA result of the weighted UniFrac distance showed that the clustering of microbiota compositions for the three groups was significantly distinct (Figure 3B). The abundance coverage-based estimator (ACE) and Chao1 indexes in the HFD were larger than those in the CK and CGLC groups (*P* < 0.05), indicating a higher microbial richness in the HFD group. However, compared with the two other groups, the Shannon and Simpson indexes of the HFD showed a marked decrease (*P* < 0.01), suggesting a lower microbial diversity in the HFD group (Figure 3C).

**FIGURE 4:**
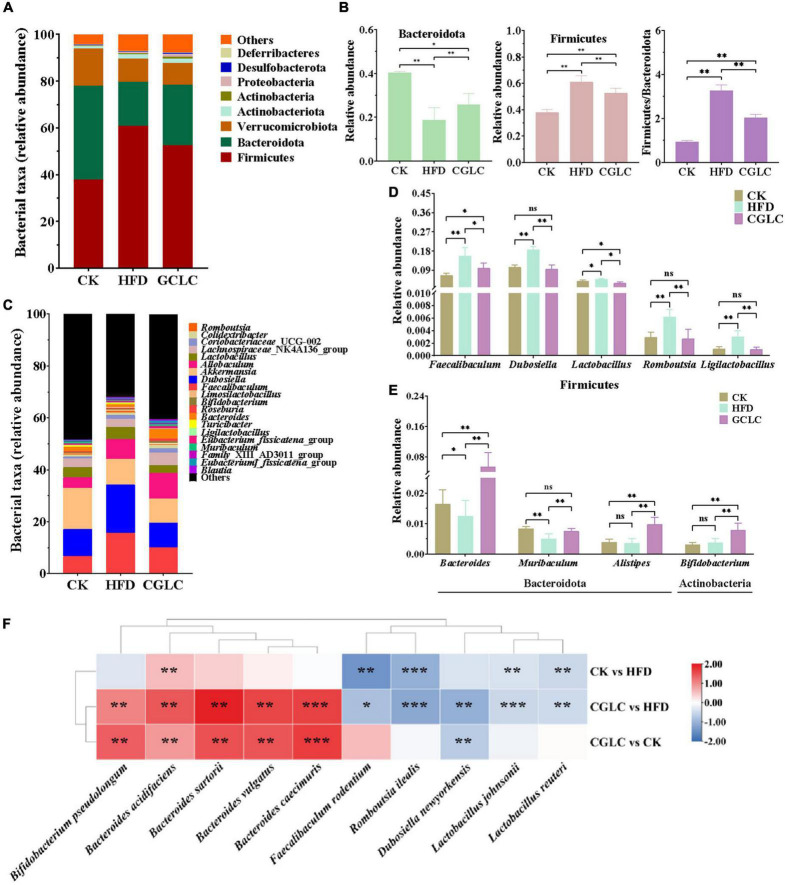
*Cordyceps guangdongensis* lipid-lowering compound formula improved the composition of gut microbiota in HFD mice. Average percent of community abundance on phylum level in three groups **(A)**. Relative community abundance of Bacteroidota and Firmicutes and Firmicutes/Bacteroidetes community abundance ratio in fecal samples of three samples **(B)**. Average percent of community abundance at the genus level in three groups **(C)**. Relative community abundance of eight genera significantly downregulated **(D)** and upregulated **(E)** in the comparison of HFD and CGP groups. **(F)** Heat map showing the difference value both of two groups (log2 fold change). ns, no significant difference; *, significant difference (*P* < 0.05), ^**^ and ^***^, highly significant difference (*P* < 0.01 and *P* < 0.001).

To further assess the effect of CGLC on the overall bacterial community overall structure, the degree analyses were further performed at the phylum, genus, and species levels. In terms of phylum level (Figure 4A), the predominant bacterial phylum in the CK group was Bacteroidota, followed by Firmicutes and Verrucomicrobiota; however, Firmicutes was the predominant bacterial phylum in the HFD group, followed by Bacteroidota and Verrucomicrobiota (Figures 4A,B). Compared to the HFD group, after 14-week intervention of CGLC, the relative abundance of Bacteroidota in the CGLC group showed a dramatical increase, whereas Firmicutes was downregulated. In addition, the ratio between Firmicutes and Bacteroidota in the CGLC group decreased 37.32% of that in the HFD group (Figure 4B). These results suggested that high-fat diet changed dominant taxa from Bacteroidota to Firmicutes, and CGLC alleviated the shift.

The top 20 taxa at the genus level were further analyzed in Figure 4C. Among them, *Faecalibaculum*, *Akkermansia*, *Dubosiella*, *Lactobacillus*, and *Bacteroides* were the predominant genera. Compared to the CK group, *Dubosiella* and *Faecalibaculum* in the HFD group showed 76.74 and 133.50% increases, respectively; in addition, the relative abundances of *Romboutsia*, *Ligilactobacillus*, and *Lactobacillus* of Firmicutes in the HFD group also showed highly obvious increases (Figure 4D, *P* < 0.01). However, the relative abundances of *Muribaculum* and *Bacteroides* showed 39.85 and 24.85% decreases (Figure 4E). After 14-week intervention of CGLC, the relative abundances of *Ligilactobacillus*, *Romboutsia*, *Dubosiella*, *Faecalibaculum*, and *Lactobacillus* of Firmicutes significantly decreased by 66.59, 55.79, 48.47, 35.77, and 35.70%, respectively; nevertheless, a significant increase was found for the relative abundances of *Bacteroides*, *Alistipes*, *Bifidobacterium*, and *Muribaculum*. Moreover, the top 10 differential taxa at the genus level were analyzed (Figure 4F). In the comparison CK vs HFD, the relative abundances of *Faecalibaculum rodentium*, *Lactobacillus johnsonii*, *Romboutsia ilealis*, and *Lactobacillus reuteri* in the HFD group showed a highly significant upregulation (*P* < 0.01), while *Bacteroides acidifaciens* in the HFD group showed an obvious decrease (32.64%, *P* < 0.01). After 14-week administration of CGLC, the relative abundances of *Bacteroides sartorii*, *B. acidifaciens*, *B. caecimuris*, *B. vulgatus*, and *Bifidobacterium pseudolongum* showed a markedly significant increase (*P* < 0.01); however, *R. ilealis*, *D. newyorkensis*, *F. rodentium*, *Lactobacillus johnsonii*, and *Lactobacillus reuteri* showed obvious decreases. These results indicated that CGLC treatment increased the relative abundances of *Bacteroides* and *Bifidobacterium* but decreased *Romboutsia* and *Dubosiella*.

### *Cordyceps guangdongensis* lipid-lowering compound formula promoted the production of short-chain fatty acids by gut microbiota in high-fat diet mice

**FIGURE 5:**
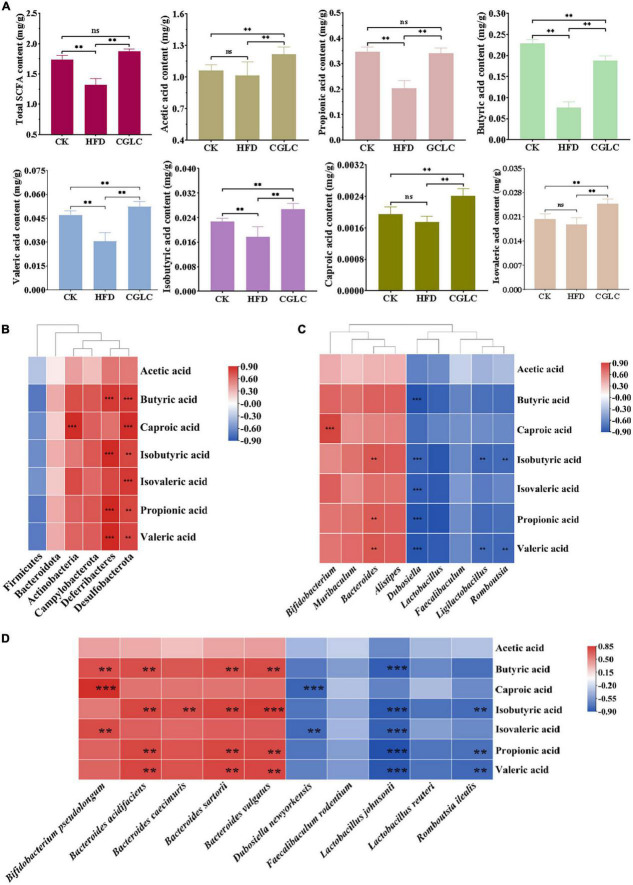
*Cordyceps guangdongensis* lipid-lowering compound formula increased the contents of SCFAs in HFD mice **(A)**. Association analysis between the abundance of gut microbiota at the phylum level and SCFAs **(B)**. Association analysis between the abundance of gut microbiota at the genus level and SCFAs **(C)**. Association analysis between the abundance of gut microbiota at the species level and SCFAs **(D)**. ns, no significant difference; ^**^ and ^***^, significant correlation (*P* < 0.01 and *P* < 0.001).

Considering that SCFAs can affect the absorption of sugars and energy, the fecal samples from three group mice were collected for SCFA content measurement. As shown in Figure 5A, a high-fat diet significantly reduced the total SCFA production with an approximate 21.06% decrease compared to the CK group (*P* < 0.01). After 14-week administration of CGLC, the total SCFA content of the CGLC group showed a marked increase (about 26.27%, *P* < 0.01) and had no obvious difference compared to the CK group. In terms of certain SCFA, the production of propionic acid (PA), butyric acid (BA), valeric acid (VA), and isobutyric acid (IBA) reduced in the HFD group (*P* < 0.01). Nevertheless, a 14-week gavage of CGLC significantly promoted the production of all the SCFAs of obese mice. Among them, the contents of acetic acid (AA), VA, IBA, isovaleric acid (IVA), and caproic acid (CA) in the CGLC group were markedly higher than those of the CK group (*P* < 0.01), whereas the butyric acid content in CGLC group showed a significant decrease.

Changes in gut microbiota composition can likewise regulate the production of SCFAs. Therefore, the association analysis of gut microbiota and SCFAs was performed. At the phylum level, Desulfobacterota had an positive correlation with the production of SCFAs except AA; the production of BA, IBA, PA, and VA showed a significantly positive correlation with Deferribacteres (*P* < 0.01); a markedly positive correlation was found between Actinobacteria and CA (Figure 5B, *P* < 0.001). At the genus level (Figure 5C), the production of IBA and PA and VA positively correlated with *Bacteroides* (*P* < 0.01), and a highly positive correlation existed between CA and *Bifidobacterium* (*P* < 0.001). However, *Dubosiella* markedly negatively correlated with BA, IBA, IVA, PA, and VA (*P* < 0.001). In addition, the production of IBA and VA showed a significantly negative correlation with *Ligilactobacillus* and *Romboutsia* (*P* < 0.01). At the species level, *Bacteroides acidifaciens* and *B. sartorii* and *B. vulgatus* showed significantly positive correlations with BA, IBA, PA, and VA (*P* < 0.01), *Bifidobacterium pseudolongum* positively correlated with BA and CA and IVA (*P* < 0.01), and a markedly positive correlation was found between *Bacteroides caecimuris* and IBA (Figure 5D, *P* < 0.01). Nevertheless, *Lactobacillus johnsonii* significantly negatively correlated with BA, IBA, IVA, PA, and VA (*P* < 0.001), and a highly negative correlation was observed between *D. newyorkensis* and CA and IVA (*P* < 0.01). In addition, we found that *R. ilealis* showed a markedly negative correlation with IBA, PA, and VA (*P* < 0.01). The above findings suggest that CGLC can promote the production of SCFAs by increasing the abundance of *Bacteroides* and *Bifidobacterium* or decreasing the abundance of *Dubosiella* and *Ligilactobacillus* and *Romboutsia*, further regulating the absorption of sugars and energy.

### Association analysis of serum lipid indicators and gut microbiota and short-chain fatty acids

**FIGURE 6:**
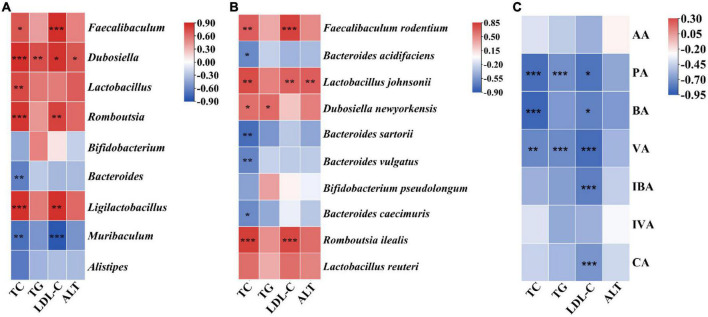
Association analysis of biochemical parameters of serum and gut microbiota at the genus level **(A)**. Association analysis of biochemical parameters of serum and gut microbiota at the species level **(B)**. Association analysis of biochemical parameters of serum and SCFAs **(C)**. *, significant difference (*P* < 0.05), ^**^ and ^***^, highly significant difference (*P* < 0.01 and *P* < 0.001).

Spearman’s coefficient was calculated to estimate the relationship between the biochemical parameters of serum and the mainly differentially genera and SCFAs. As shown in Figure 6A, four indexes (TC, TG, LDL-C, and ALT) showed positive correlations with *Dubosiella* (*P* < 0.05); TC and LDL-C markedly positively correlated with *Faecalibaculum*, *Romboutsia*, and *Ligilactobacillus* (*P* < 0.05); and TC and *Lactobacillus* were positively correlated (*P* < 0.01). However, TC significantly negatively correlated with *Bacteroides* and *Muribaculum* (*P* < 0.01), and a highly markedly negative correlation was obtained between LDL-C and *Muribaculum* (*P* < 0.001). At the species level, it was found that TC showed an positive correlation with *F. rodentium*, *Lactobacillus johnsonii*, *D. newyorkensis*, and *R. ilealis* (*P* < 0.05); LDL-C highly positively correlated with *F. rodentium*, *Lactobacillus johnsonii*, and *R. ilealis* (*P* < 0.01); TG only had a positive correlation with *D. newyorkensis* (*P* < 0.05); ALT and *Lactobacillus johnsonii* were significantly positively correlated with each other (Figure 6B, *P* < 0.01). In addition, negative correlations were found between TC and *Bacteroides sartorii*, *B. vulgatus*, *B. caecimuris*, and *B. acidifaciens* (*P* < 0.05). The association analysis of biochemical parameters of serum and SCFAs found that PA and VA had an negative correlation relationship with TC, TG, and LDL-C (*P* < 0.05); BA markedly negatively correlated with TC and LDL-C; and LDL-C showed a highly significant correlation relationship with IBA and CA (Figure 6C, *P* < 0.001). Those descriptions indicate that CGLC can improve blood lipid level in serum by the abundance change in gut microbiota and the increase in SCFA production.

### *Cordyceps guangdongensis* lipid-lowering compound formula affected mRNA expressions of genes involved in lipid and fat metabolism

**FIGURE 7:**
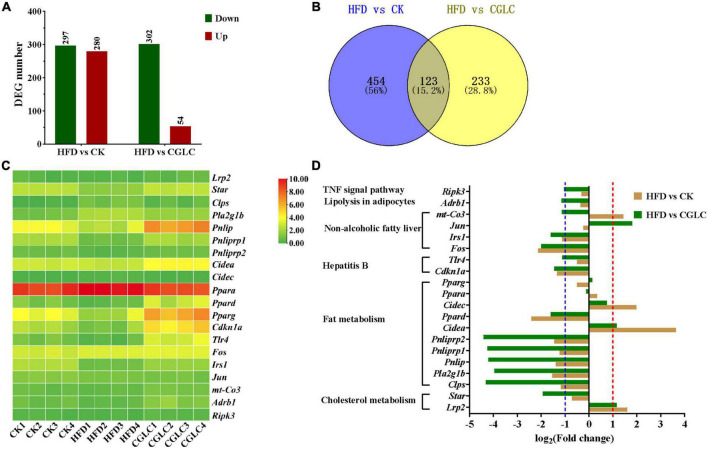
Number distribution of DEGs in HFD vs CK and HFD vs CGLC **(A)**. Venn map showing DEG overlapping of two comparisons **(B)**. Heat map showing the log_2_(FPKM + 1) values of genes involved in fat and lipid metabolism in three group **(C)**. Fold changes of genes involved in fat and lipid metabolism in two comparisons **(D)**.

To explore the expression pattern of genes involved in lipid and fat metabolism, liver samples from three group were collected and used for transcriptome analysis. Compared to the HFD group, 577 DEGs (297 upregulation and 280 downregulation) and 356 DEGs (302 upregulation and 54 downregulation) were obtained in the CK and CGLC groups, respectively (Figure 7A and Supplementary Tables 2, 3). A total of 123 DEGs were shared in HFD vs CK and HFD vs CGLC (Figure 7B). Among these overlapping DEGs, 20 genes were related to fat and lipid metabolism. According to the RPKM values, it was found that their expression patterns in the CGLC group were similar to those of the CK group (Figure 7C).

Among 10 genes involved to fat metabolism, six genes (colipase pancreatic *Clps*, phospholipase group IB pancreas *Pla2g1b*, pancreatic lipase *Pnlip*, pancreatic lipase-related protein 1 *Pnliprp1*, *Pnliprp2*, and peroxisome proliferator activator receptor delta *Ppard*) were markedly upregulated in the comparison CGLC vs HFD, whereas the expression levels of those genes were obvious downregulation or showed no significant differences for HFD vs CK; nevertheless, the expression levels of cell death-inducing DNA fragmentation factor alpha subunit-like effector A *Cidea* and cell death-inducing DFFA-like effector c *Cidec* showed marked upregulation only in the HFD group (Figure 7D and Supplementary Table 4).

In terms of two genes related to cholesterol metabolism, *Lrp2* (low-density lipoprotein receptor-related protein 2) was upregulated in the HFD group, whereas the expression level of *Star* (steroidogenic acute regulatory protein) showed significant upregulation in the CGLC group (Figure 7D). For six genes related to hepatitis B and NAFLD, *Cdknla* (cyclin-dependent kinase inhibitor 1A), *Tlr4* (toll-like receptor 4), *Fos* (FBJ osteosarcoma oncogene), *Jun* (jun proto-oncogene), and *Irs1* (insulin receptor substrate 1) in the CGLC group were upregulated compared to the HFD group, whereas *Cdknla* and *Fos* and *Irs1* showed an obvious downregulation in the comparison HFD vs CK (Figure 7D and Supplementary Table 4). In addition, receptor-interacting serine–threonine kinase 3 (*Ripnk3)* and adrenergic receptor beta 1 (*Adrb1)* were upregulated only in the comparison CGLC vs HFD (Figure 7D and Supplementary Table 4). These above-mentioned results suggest that CGLC administration can regulate the expression levels of genes related to lipid and fat metabolism in obese mice induced by high-fat diet, further decreasing the fat accumulation in liver.

## Discussion

In the last decade, gut microbiota has become a hot research topic and shown as a crucial regulator for substrate metabolism, energy balance, and health status in organisms (35–38). A growing number of reports documented that many chronic diseases (e.g., obesity and T2MD) were associated with gut microbiota dysbiosis (39–41), and TCM played an important and positive role in improving obesity-related metabolic diseases (20–22, 42). In this current study, we investigated that the effects of CGLC intake on fat accumulation, lipid metabolism, gut microbiota, SCFA production, and expression levels of genes related to fat and lipid metabolism in obese mice were induced by high-fat diet.

Consistent with anti-obesity effect of single ingredient from CGLC, we found that CGLC significantly reduced fat accumulation, hyperlipidemia, and glucose tolerance in mice with a HFD diet (Figures 1A–C, 2A–E), suggesting the prevention and treatment effect of CGLC against glucose and lipid metabolism disorders (19, 21, 23, 29). Compared with one of *C. guangdongensis* and *P. vulgaris* extracts, CGLC showed further improved glucose tolerance, fat metabolism, and hyperlipidemia symptom (19, 23). In addition, 14-week administration of CGLC decreased fat vacuole injury level and lipid accumulation in the liver. The liver is very crucial for lipid metabolism and lipogenesis (43), and some antiobesonenic candidates were reported to prevent obesity and serum lipid levels by regulating fat and lipid metabolism in the liver (44). The expression levels of Pnliprp, PPARs, and Cide protein family participated in the regulation of lipid digestion and metabolism, adipogenesis and adipocyte differentiation, lipid oxidation, and lipid droplet formation (45–48). Our transcriptome results showed that after CGLC administration of 14 weeks, *Pnliprp1*, *Pnliprp2*, and *Ppard* were markedly upregulated, whereas the expression levels of *Cidea* and *Cidec* showed an obvious downregulation (Figure 7D), being consistent with the effect of *Moringa oleifera* leave crude polysaccharide on the expression pattern of those genes (49). From the perspective of cholesterol metabolism, *Lrp2* (low-density lipoprotein receptor-related protein 2) was downregulated in the CGLC group, indicating a low level of LDL-C (50). In addition, genes involved into NAFLD showed a significant upregulation in the comparison CGLC vs HFD, which is consistent with the decreasing of fat vacuole injury level and lipid accumulation in the liver after 14-week administration of CGLC.

Gut microbiota plays a crucial role in the maintenance of dyslipidemia, T2MD, and NAFLD (51, 52). HFD greatly affected the abundance of gut microbiota at the phylum and genus levels, whereas CGLC restored that imbalanced change (Figure 4), indicating that CGLC might intervene the occurrence of metabolic disorders through regulating gut microbiota. Bacteroidota is well-known probiotics that could suppress obesity-related disease occurrence (39), but Firmicutes have been reported to be positively correlated with the onset of obesity-related diseases (53). The administration of CGLP significantly decreased the abundance of Firmicutes but increased the abundance of Bacteroidota at the phylum level, being consistent to the link between obesity and the abundance changes of Firmicutes and Bacteroidota (19, 54, 55). Many obesity-associated metabolic phenotypes were correlated with *Bacteroides* such as *B. uniformis* and *B. caccae*, and *B. acidifaciens* can prevent obesity and improve insulin sensitivity in mice (56, 57). Some bacteria from *Alistipes* are likely to provide energy to the host by promoting energy harvesting (58). Supplementation of *Bifidobacterium pseudolongum* markedly decreased body mass and visceral fat and plasma triglycerides and increased the colonization of beneficial bacteria (56). *Muribaculu*m has been observed to be negatively correlated with metabolism disorder (59). After 14-week CGLC gavage, we found that the relative abundances of *Bacteroides* (*B. acidifaciens*, a gut commensal bacterium preventing obesity and improving insulin sensitivity), *Alistipes*, *Bifidobacterium* (*Bifidobacterium pseudolongum*), and *Muribaculum* showed an obvious upregulation (Figure 4E), and *Bacteroides* and *Muribaculum* significantly negatively correlated with the levels of TC and LDL-C (Figure 6A). Nevertheless, the relative abundances of *Romboutsia*, *Dubosiella*, and *Faecalibaculum* were significantly downregulated in the CGLC group (Figure 4D), and they showed positive correlations with serum lipid indicators, being consistent with the increase in their abundances in the obese mice according to previous studies (49, 60, 61).

SCFAs, bioconversion products of fermentable carbohydrates by gut microbiota, play a crucial intermediate role in connecting the body health and intestinal microorganisms (62, 63). The contents of AA, PA, and BA accounted for 91.18% of SCFAs in the gut and showed a marked increase after 14 weeks of CGLC administration (Figure 5A). These three SCFAs can lower the gut pH value, suppress the growth of harmful bacteria, and increase the number of beneficial bacteria. In addition, they improved obesity-related diseases by regulating gluconeogenesis pathway, reducing inflammation, and maintaining gut homeostasis (5, 64). In our model, AA content in the CGLC group significantly increased and showed a similar trend with the relative abundances of *Bacteroides* and *Alistipes* which produce AA as a major product of carbohydrate fermentation (65–67). The CA showed a markedly positive correlation with *Bifidobacterium*, and *Bacteroides* significantly positively correlated with IBA, PA, and VA. Nevertheless, BA and PA showed an negative correlation relationship with *Dubosiella*, and *Romboutsia* markedly negatively correlated with IBA and VA (Figure 5C). The association analysis of serum lipid indicators and SCFAs indicated three serum lipid indexes negatively correlated with PA, BA, VA, IBA, and CA (Figure 6C). Furthermore, we speculated that CGLP can affect the change in gut microbiota composition and further regulate the production of SCFAs to mediate lipid metabolic disorder, which has been proved by transplanting fecal microbiota into obese mice (68–70).

In summary, CGLC administration significantly improved hyperlipidemia and glucose tolerance, reduced fat accumulation in body, and decreased lipid droplet accumulation in the liver by modulating gut microbiota, SCFAs, and expression level of genes related fat and lipid metabolism. Therefore, our findings indicate that CGLC has the potential as one dietary intervention method for alleviating obesity-related diseases in future.

## Data availability statement

The original contributions presented in this study are included in the article/Supplementary material, further inquiries can be directed to the corresponding authors.

## Ethics statement

This animal study was reviewed and approved by Animal Care and Use Committee of Institute of Microbiology, Guangdong Academy of Sciences (No. GT-IACUC20210406).

## Author contributions

GW, CS, CZ, TL, and WD conceived and designed the experiments. CS and GW performed the experiments. GW, CS, XC, CZ, and QH collected all the experimental samples. GW, CS, and TW analyzed the data. GW, CS, and BX observed the characteristics of tissue samples. GW wrote the manuscript. WD and HL reviewed the manuscript. All authors contributed to the article and approved the submitted version.
